# Supplementary material for: Larval development and survival of pond-breeding anurans in an agricultural landscape impacted more by phytoplankton than surrounding habitat
Source: PLoS One. 2021 Jul 26;16(7):e0255058. doi: 10.1371/journal.pone.0255058 (PMC8312938; doi:10.1371/journal.pone.0255058)
Supplement: Attachment — Submitted filename: Response_To_Reviewers.docx [file pone.0255058.s001.docx]

**Department of Biology**

**212 Pearson Hall**

**Oxford, OH 45056**

14 May 2021

Dear Dr. Ji-Zhong Wan:

We have revised the manuscript now titled “Larval development and survival of pond-breeding anurans in an agricultural landscape impacted more by phytoplankton than surrounding habitat” (PONE-D-21-04128) and believe our manuscript has substantially improved thanks to the reviewer insights. We have attached a manuscript using track changes, and the line numbers we reference below refer to the track-changes version, as well as a “clean” revision. We document our changes in the letter below in red font, following reviewer comments.

We examined how surrounding habitat (forest, grassland, and agriculture) adjacent to created ponds affected anuran larval development in an *in situ* field study and we followed one species into the terrestrial environment to look for subsequent carryover effects. We found that two species of anurans survived and developed in ponds regardless of the surrounding habitat and that growth was positively influenced by phytoplankton abundance (an estimate of food availability), which varied more among ponds than among habitat types. Size at the end of the aquatic size was predictive of overwinter survival in terrestrial habitats, highlighting the importance of aquatic conditions across life stages. Overall our work highlights the value of created ponds to at risk taxa on the landscape, and points to the critical linkage between aquatic and terrestrial habitats in evaluating land use/land cover impacts on amphibians.

Thank you for the opportunity to revise.

Sincerely,

Michelle D. Boone, Ph.D.	Melissa B. Youngquist, Ph.D.

Associate Professor	Research Biologist, Shedd Aquarium

PONE-D-21-04128
Independent of surrounding terrestrial habitat, human-made ponds create valuable habitat for pond-breeding anurans in agricultural landscapes
PLOS ONE

Dear Dr. Boone,

Thank you for submitting your manuscript to PLOS ONE. After careful consideration, we feel that it has merit but does not fully meet PLOS ONE’s publication criteria as it currently stands. Therefore, we invite you to submit a revised version of the manuscript that addresses the points raised during the review process.

Please submit your revised manuscript by 16th, May, 2021. If you will need more time than this to complete your revisions, please reply to this message or contact the journal office at [plosone@plos.org](mailto:plosone@plos.org). When you're ready to submit your revision, log on to <https://www.editorialmanager.com/pone/> and select the 'Submissions Needing Revision' folder to locate your manuscript file.

Please include the following items when submitting your revised manuscript:

- A rebuttal letter that responds to each point raised by the academic editor and reviewer(s). You should upload this letter as a separate file labeled 'Response to Reviewers'.
- A marked-up copy of your manuscript that highlights changes made to the original version. You should upload this as a separate file labeled 'Revised Manuscript with Track Changes'.
- An unmarked version of your revised paper without tracked changes. You should upload this as a separate file labeled 'Manuscript'.

If you would like to make changes to your financial disclosure, please include your updated statement in your cover letter. Guidelines for resubmitting your figure files are available below the reviewer comments at the end of this letter.

If applicable, we recommend that you deposit your laboratory protocols in [protocols.io](http://protocols.io/) to enhance the reproducibility of your results. Protocols.io assigns your protocol its own identifier (DOI) so that it can be cited independently in the future. For instructions see: <http://journals.plos.org/plosone/s/submission-guidelines#loc-laboratory-protocols>. Additionally, PLOS ONE offers an option for publishing peer-reviewed Lab Protocol articles, which describe protocols hosted on [protocols.io](http://protocols.io/). Read more information on sharing protocols at <https://plos.org/protocols?utm_medium=editorial-email&utm_source=authorletters&utm_campaign=protocols>.

We look forward to receiving your revised manuscript.

Kind regards,

Ji-Zhong Wan
Academic Editor
PLOS ONE


Additional Editor Comments:

Please revise the manuscript according to the reviewers' comments fully.

Journal Requirements:

When submitting your revision, we need you to address these additional requirements.

1. Please ensure that your manuscript meets PLOS ONE's style requirements, including those for file naming. The PLOS ONE style templates can be found at

<https://journals.plos.org/plosone/s/file?id=wjVg/PLOSOne_formatting_sample_main_body.pdf> and

https://journals.plos.org/plosone/s/file?id=ba62/PLOSOne_formatting_sample_title_authors_affiliations.pdf

2. In your Methods section, please provide additional information regarding the permits you obtained for the work. Please ensure you have included the full name of the authority that approved the field site access and, if no permits were required, a brief statement explaining why.

This information is all included under the “Ethics statement” at the beginning of the Methods.

3. In your Methods section, please include a comment about the state of the animals following this research. Were they euthanized or housed for use in further research? If any animals were sacrificed by the authors, please include the method of euthanasia and describe any efforts that were undertaken to reduce animal suffering.

This information is all included under the “Ethics statement” at the beginning of the Methods.

4. We note that the grant information you provided in the ‘Funding Information’ and ‘Financial Disclosure’ sections do not match.

When you resubmit, please ensure that you provide the correct grant numbers for the awards you received for your study in the ‘Funding Information’ section.

 The funding for the ASG (Amphibian Specialist Grant) grant was awarded to MBY, but it does not have a grant number.

5. We note that you have stated that you will provide repository information for your data at acceptance. Should your manuscript be accepted for publication, we will hold it until you provide the relevant accession numbers or DOIs necessary to access your data. If you wish to make changes to your Data Availability statement, please describe these changes in your cover letter and we will update your Data Availability statement to reflect the information you provide.

This is a private link, which we will publish upon acceptance: <https://figshare.com/s/98a91b49a60114a94c36>

Reviewers' comments:

Reviewer's Responses to Questions

**Comments to the Author**
1. Is the manuscript technically sound, and do the data support the conclusions?

The manuscript must describe a technically sound piece of scientific research with data that supports the conclusions. Experiments must have been conducted rigorously, with appropriate controls, replication, and sample sizes. The conclusions must be drawn appropriately based on the data presented.

Reviewer #1: Yes

Reviewer #2: Yes

2. Has the statistical analysis been performed appropriately and rigorously?

Reviewer #1: Yes

Reviewer #2: Yes

3. Have the authors made all data underlying the findings in their manuscript fully available?

The [PLOS Data policy](http://www.plosone.org/static/policies.action#sharing) requires authors to make all data underlying the findings described in their manuscript fully available without restriction, with rare exception (please refer to the Data Availability Statement in the manuscript PDF file). The data should be provided as part of the manuscript or its supporting information, or deposited to a public repository. For example, in addition to summary statistics, the data points behind means, medians and variance measures should be available. If there are restrictions on publicly sharing data—e.g. participant privacy or use of data from a third party—those must be specified.

Reviewer #1: No

Reviewer #2: Yes

We have updated this information in figshare and the link is here: <https://figshare.com/s/98a91b49a60114a94c36> , currently private, but we will make it public after acceptance.

4. Is the manuscript presented in an intelligible fashion and written in standard English?

PLOS ONE does not copyedit accepted manuscripts, so the language in submitted articles must be clear, correct, and unambiguous. Any typographical or grammatical errors should be corrected at revision, so please note any specific errors here.

Reviewer #1: Yes

Reviewer #2: Yes

5. Review Comments to the Author

Reviewer #1: The manuscript by Youngquist and Boone is a nice work showing that human-made ponds that exhibit great variation in phytoplankton biomass and that have a vegetation buffer are valuable habitats for pond-breeding amphibians, regardless of their terrestrial surroundings. Unfortunately, the small number of ponds analyzed restrict the conclusions to ponds subject to the specific conditions as those analyzed (with a surrounding buffer area and high environmental variability). These limitations, however, are all well addressed in the discussion. I just feel that some sentences in the conclusions should be a bit more linked to these limitations (see specific comments). It is hard to say from these results that human-made ponds in agricultural fields are as good as those in any other terrestrial matrix. But we can say that other factors might be more important (but we do not know why exactly they are varying here). That being said, this is still valuable work, done with great care. The authors seemed to have handled well all the unexpected difficulties inherent to any experimental approach, especially regarding field experiments. The whole paper is generally well written. The introduction builds logically to the hypotheses tested, and the discussion, as already said, acknowledges the limitations of the experimental design and draws conclusions based on the results. The methods and results are generally good but could use a bit more details on some aspects of the methodology and the variability in environmental conditions found between ponds. I believe the figures can also be improved. Furthermore, I believe all of these issues are addressable. See specific comments below:

#1 We agree that more ponds would have been ideal, and the number was limited by practical constraints. We have found effects in other experiments with a similar number of ponds between reference and golf course ponds (4 ponds total; 2 reps; Boone et al. 2008) and between golf course ponds with and without a terrestrial buffer (6 ponds total; 3 reps; Puglis & Boone 2012), however; so if there were strong differences associated with land-use/land-cover, we could see differences with the current level of replication (2-3 ponds per habitat type). We have kept this comment in mind during revision and have edited the text to highlight this (For example, See Lines 344-361 [in “Revised_Manuscript_With_Track_Changes” for all line references]).

We have revised the text throughout to deemphasize the lack of a habitat effect per se, and highlighted the sample size may obscure land use/land cover effects when there is high variation and many other factors influencing habitat quality (e.g., Lines 354-361, 468-473).

We have revised the figures for clarity and added the requested details in the methods.

Introduction
Lines 90 - 91 – What is the difference expected between the two species?

#2 We clarified that we predicted that cricket frogs would be more sensitive to ponds based on surrounding habitat than bullfrogs based on cricket frog declines and bullfrogs expanding ranges (Lines 101-102, 339-340).

Methods
Line 115 – Is the city water treated with chlorine or any chemical compounds that could be harmful in any way or change the behavior of the tadpoles?

#3 We clarified in the text when pond mesocosms were set up (Lines 129-130).

Lines 116 – 117 - Tadpoles were maintained in mesocosms for how much time until they were transferred to the in situ enclosures? I know the reader could do the math, but it's easier if you explicitly give the information here.

#4 Cricket frogs and bullfrogs were all recently hatched when added to the enclosures. We updated this information in the text; eggs/tadpoles were held for 15-16 days before being added to enclosures, and then approximately 12 days later, some enclosures were replaced with individuals from enclosures. (See Lines 170-179)

Line 129 - Is the presence of fish common among these constructed ponds? Are they introduced by landowners? Or do they colonize ponds naturally after some time?

#5 We clarified that fish had been stocked in ponds by landowners at some point in the past (See Line 145).

Lines 134 – 135 - Do you have any information on what kind of fertilizers and pesticides are used in those different crops? More importantly, do you know when they are usually applied? Some crops heavily rely on insecticides while others rely more on herbicides and they may have quite different consequences to amphibians as shown in:
Relyea RA. The lethal impact of Roundup on aquatic and terrestrial amphibians. Ecological Applications. 2005;15: 1118–1124. doi:10.1890/04-1291
Relyea RA, Schoeppner NM, Hoverman JT. Pesticides and amphibians: The importance of community context. Ecological Applications. 2005;15: 1125–1134. doi:10.1890/04-0559

#6 Herbicides and fertilizers are the most common chemical application, but we do not know the history of use in the sites that we used. However, you will note that ponds in each habitat type had a range of low to high survival, so it would be difficult to attribute effects to contamination. We did not test for pesticides, because of cost constraints, but we did measure nutrients and water quality—even these variables showed wide variation among sites. We have included mean + SE of each parameter in the methods in the revision (See Lines 271-274).

Lines 146 – 147. Do you have pictures of each of those ponds and the enclosures to add as supplements? I often like to see pictures to have a better idea of the study system.

#7 We have included a picture of ponds and enclosures from each habitat type as a figure in the revised draft, which could be moved to a supplementary figure. (See Fig 1)

Lines 152 – 155. Did these new tadpoles also enter the experiment in stage 25? From what habitat types were those enclosures? Were they all aggregated in a single habitat? Single pond? Were they evenly distributed across ponds and habitats? This is important.

#8 We have added this information to the text: that tadpoles were still at Gosner stage 25-26, as well as the specific number of enclosures replaced in which ponds. (See Lines 172-179)

Lines 156 – 158. Did you test the water for pesticides?

#9 Because of cost constraints (~$150/sample for a specific pesticide) and because it would be difficult to attribute effects to pesticides given the number of ponds, we did not sample for pesticides across our 9 sites.

Lines 181 – 182. “we calculated percent survival and average mass, time to metamorphosis, and Gosner developmental stage”. You already said that at the beginning of the paragraph.

#10 We deleted this text.

Lines 182 – 183. “Percent survival and mass for both species were transformed to meet the assumption of normality”. What kind of transformation?

#11 We clarified that we arcsine square root and log transformed proportion survived and mass, respectively. (See Lines 208-209)

Lines 189 – 191. Any guesses on the reason for these high levels of nutrients?

#12 The pond was in an agricultural landscape, and direct application to nearby ponds may have negatively impacted this pond, but we did not speculate because the reason is not clear. We did include information indicating that this was an agricultural pond. (See Lines 216-220)

Lines 196 – 198 - I am worried that you might be losing some relevant information by averaging all your values over time. Did you look at the variances over time for each habitat type as well? Maybe some habitat types exhibit greater variation in water quality variables over time, and that may be detrimental to tadpoles. Agricultural fields usually receive pesticide and fertilizer pulses in specific moments of their crop cycles. This may cause water parameter values to change drastically and then return to regular levels. If that happened, it could not show up as clearly in the mean as it would in the variance.

#13 We did analyze the response of water quality variables by habitat over time, but there were not significant treatments effects likely because the variation among the ponds did not appear to be associated with habitat. To get a value with which to test the relationship between water quality and tadpole responses, we elected to use the mean, which we thought was a reasonable estimate of the endpoint. We could have looked at each endpoint at a single time measurement, but that did not seem appropriate given that it substantially increased the number of statistical tests when we had a relatively small sample size. For the variables below, we report mean and SE. Most of the variation is between rather than within ponds over time.

Lines 202 – 204. If you tested these variables separately, you do not have to exclude any of them because of multicollinearity. Unless you want to avoid making too many statistical tests (reducing your overall statistical power).

#14 This is a fair point. We were attempting to reduce the number of statistical tests to reduce the likelihood of a Type I error. We have rephrased this in the text (Line 223-226).

Lines 215 – 216. Again, it would be nice to see pictures as supplements.

See Comment #7 above

Lines 224 – 225 “Enclosures were checked weekly until no more animals were encountered. 225 (19 April 2014)”. I did not understand this. Why were they not encountered? Did they die? Even the ones that “survived” overwinter? Did they escape? Or do you mean the enclosures from the first experiment?

#15 We have edited this sentence for clarity. (See Lines 254-256)

Lines 229 – 230. What statistical distribution (or family) did you use in your generalized linear models?

#16 We used a binomial distribution to examine the effect of initial mass on survival and a Gaussian distribution to examine the effect of initial mass on overwinter growth. We clarified this in the text. (See Line 262)

Lines 230 – 231. I believe it would best to use the terrestrial enclosure as a random effect in a mixed model approach (maybe even the aquatic one too but could make the model too complex for the sample size and it may not converge). You can do that in a generalized linear mixed model. Packages lme4 and glmmTMB do that in R.

#17 In preliminary analyses, we tried nesting enclosure in pond as a random effect, but the model did not converge. Additionally, when using terrestrial enclosure as a random effect in a GLMM to test initial mass, the model did not converge. Therefore, we used "pond" as the random effect because we were testing the effect of "habitat" with "pond" as the replicate and used a GLM. When testing pond as a fixed effect, we didn't see any difference in results when using "enclosure" as a random factor in GLMM vs allowing them to be replicates in a GLM.

Results
Line 238 – “(F < 2.21, P > 0.147)”. Aren’t those exact F and P values? If so (even if they were rounded to fewer decimal digits) you can use an = sign instead of < or >.

#18 When there were multiple metrics that were not significant, we indicated the highest F-statistics (and the lowest P-value) to demonstrate that variables were not affected by habitat type.

Lines 243 – 246. Even though it was not "truly" significant, I think you can also highlight this result in table 2 (Phytoplankton and bullfrog variables). Maybe in italic to differentiate from the ones that were significant even after correction.

#19 We made this change. (See Table 2)

Lines 271 – 273. It is odd that pond affected mass at metamorphosis, initial mass (after metamorphosis) affected overwinter survival, but pond did not affect overwinter survival. Do you know why? Poor statistical power perhaps?

#20 The lack of a pond effect on terrestrial responses likely occurs for a couple of reasons, including low power to detect pond differences. However, while there were pond effects on mass at metamorphosis (from a total of eight ponds), cricket frogs from only two ponds from each habitat type (a total of six ponds) were followed in terrestrial enclosures (because of low survival from one pond in each habitat); this may also contributes to lack of an effect. (See Lines 413-421)

Lines 274. “final mass (F1,46 = 12.82, P = 0.0008), and change in mass (F1,46 = 4.20, P = 0.0462)”. Why not show this in figure 4 as well?

#21 We agree and we made this change, now Fig 5.

Figures. For all figures. The font size of the axis labels is too small, maybe in a formatted paper, they will be difficult to read.

#22 We revised all figures and increased font size.

Figure 1. The way results are being shown here is odd. I understand the authors wanted to show ponds individually, but, it REALLY looks like there are three treatments (white, gray, and black) that are very different from each other. But this treatment does not exist. It is nearly impossible to look at the figure and not to think about that. On the other hand, it is not clear to me what would be the best solution for this. You could mix the colors a bit more, so black, gray, and white symbols do not stay together at the top or bottom of the graph. You could try to use different symbols for each pond (diamonds, stars, crosses, etc). Another option would be to apply different shades of gray that correspond to some important water variable, like Chlorophyll-a.

#23 We have revised the figures so that each pond has its own shape and shading.

Discussion
Lines - 345 – 347 – “The lack of an overall pond effect, despite differences in size at metamorphosis between ponds, was surprising. However, there was overlap in the distribution of initial metamorph sizes among ponds.”. Indeed! I think this deserves more elaboration.

#24 We expanded this text slightly (See Lines 413-424)

Lines – 362. “Within the Midwest”. What Midwest? I guess it is the Midwest of the USA, but it may not be clear to all readers.

#25 We have revised to read within the midwestern United States. (See Line 438)

Lines 363 - 366. Even though it has been discussed above and the information is present below, I think it should also be acknowledged here that this conclusion may be conditioned to some conditions, such as the presence of a vegetation buffer and great variability in resource availability caused by other factors. I believe you could rewrite the paragraph to strengthen the link between the conclusion and their restrictions.

#26 We added a new conclusion paragraph to the revision and kept this comment in mind during the. We also elaborated on these issues in the Discussion. (See Lines 345-361; Lines 466-477)

Reviewer #2: The authors present a field study assessing the influence of terrestrial land-use on larval survival of two amphibian species. Albeit there are some potential sample size issues and variability within land-use type that may hinder their ability to draw strong conclusions, the authors acknowledged these issues and made stronger inferences, where possible. The authors conclude that the best predictor of larval survival was phytoplankton abundance, regardless of surrounding habitat type. This paper is well-written and will be a nice contribution to the field of amphibian conservation.

General comments:
The present study was designed, in part, to understand whether aquatic environments in human dominated landscapes might be more beneficial to a species that is thriving (the bullfrog) compared to a species with a shrinking range (cricket frog). However, this question is left unanswered. As the data collected here did not answer that question, it would be of interest for the authors to speculate in the Discussion what the answer might be or the next steps to uncover it.

#27 This reviewer makes a good point and we did note this in a few additional places in the discussion. (See Lines 339-340, 379-350, 442-444, 468-470)

In the Discussion, there are several statements that indicate the authors were surprised by the outcome of this study (Lines 297 363), yet data collected by them previously indicated cricket frog presence was not uncommon in agricultural ponds (from personal observations), are able to disperse across cropland (citation 63), and the presence of cricket frogs are not predicted by the surrounding landscape (citation 46). A deeper delve into what the differences between those studies and conclusions are vs. those that have found differences (citations 43, 44) is warranted.

#28 We removed our surprise—the reviewer is right, we really were not surprised! The discussion throughout has been revised for clarity.

The authors suggest that these created ponds can be “valuable” larval habitat for amphibians. Is there any evidence these ponds are serving as population sinks?

#29 This is a good point and we have added discussion regarding the potential of some of these sites to serve as sinks (Lines 455-457).

Given the finding that phytoplankton is the strongest predictor of tadpole success, a more thorough discussion of what controls phytoplankton abundance would be useful.

#30 We have clarified that phytoplankton abundance could have been influenced by nutrients (phosphorus), as well as increased light (See Lines 384-385).

Specific comments:
Line 28: lowercase cricket frog. Check throughout.

#31 We made this change and checked throughout.

Lines 127-128 indicate some ponds were used for irrigation. Were there any drawdowns or fluctuations in water levels?

#32 While we were told some of these ponds were used for irrigation, there were not dramatic changes in pond water level over the course of our study. (See Line 143)

Lines 136: How far were the grassland ponds from agricultural plots? How large are typical tracts in the CRP? Is it likely that there are still agricultural plots draining into these ponds indicating the potential for contaminant exposure regardless of immediate adjacent habitat?

#33 This entire landscape is surrounded by agricultural lands, so it is likely all sites have some degree of pesticide exposure through some mechanism, likely atmospheric drift. However, concentrations would be low and the anurans did well in most of these sites with the results having a plausible explanation—abundance of phytoplankton.

CRP plots vary in size, and come in and out of enrollment in the program in Ohio. The grass land ponds had a minimum distance from an agricultural area as follows: Pond 4: 50 m; Pond 5: 300 m; Pond 8: 300 m

As we noted in the methods, the habitat classification were not strictly clean: one "grass" pond was partially surrounded by "forest" and one “forest” pond had a partially grassy boarder. However, the larger landscape didn't help categorize ponds clearly either: Using % land use/land cover in a buffer as a predictor of frog performance didn't change results in preliminary analyses. It was hard to get permission to use ponds in this study, so we took what was available.

Indicate the number of cricket frog tadpoles that survived but did not metamorphose.

#34 We included this information in the text (Lines 200-202).

Lines 182-183. Part of the usefulness of generalized linear mixed models is the use of link functions. This negates the need to transform data by using the appropriate distribution, recognizing the non-Gaussian nature of some datasets. For count data such as percent survival, the log-link function should be used.

#35 We tried doing this and got error messages that it had a "singular fit" and it did not change the results. We believe the statistical test we used is appropriate for the data.

Line 227: remove comma between overwinter survival and terrestrial growth. Replace with an “and”.

#36 We made this change.

Line 228: Move “Terrestrial enclosures were the experimental unit” to the end of the following sentence describing the mixed model.

#37 We made this change.

Line 238: Replace “was” with “were”

#38 We made this change.

Lines 297-305. This paragraph feels like a lot of hedging and leaves the reader wondering what the value of this study is. Are there major flaws in the design of this study (inadequate sample size, incorrect labeling of habitat type)? Rework this paragraph to have less “howevers”. Combine this paragraph with the next paragraph so the discussion of issues surrounding the defined habitat types in this study are included in one paragraph that ends with the statements regarding what can be concluded with this study- that “created ponds in different land use contexts can provide suitable larval habitat”.

#39 We revised the first two paragraphs for clarity. (See Lines 333-361)

Lines 320: lowercase bullfrog. Check throughout.

#40 We made this change.

Line 323: insert “that” between studies and have.

#41 We made this change.

Line 348: insert “than” after less.

#42 We made this change.

Line 350: What habitats enable tadpoles to reach large sizes at metamorphosis?

#43 We revised this sentence. (See Lines 421-424)

Figures and Tables
Despite the caption indicating otherwise, it is very hard not to try to see patterns among shapes in the figures. Try switching so that the shading is representative of habitat type and choose more than three shapes to represent individual ponds so that there are not the same three shapes between any two habitat types. This way the habitat type will still be represented by shading across figures and readers will not attempt to identify meaningless patterns.

#44 This is a good idea and we revised the figures according to this suggestion.

Include description regarding shading and shapes in captions for Figures 1-3 so that the description is the same and readers don’t have to look back to understand the significance.

#45 We made this change

Table 1 also includes the results from the GLMMs, correct?

#46 Yes and we clarified the Table legend.
