# Supplementary material for: Larval development and survival of pond-breeding anurans in an agricultural landscape impacted more by phytoplankton than surrounding habitat
Source: PLoS One. 2021 Jul 26;16(7):e0255058. doi: 10.1371/journal.pone.0255058 (PMC8312938; doi:10.1371/journal.pone.0255058)
Supplement: Attachment — Submitted filename: Response_To_Reviewersb.docx [file pone.0255058.s002.docx]

**Department of Biology**

**212 Pearson Hall**

**Oxford, OH 45056**

24 June 2021

Dear Dr. Ji-Zhong Wan:

We have revised the manuscript titled “Larval development and survival of pond-breeding anurans in an agricultural landscape impacted more by phytoplankton than surrounding habitat” (PONE-D-21-04128R1) and believe our manuscript has addressed the remaining concerns of the reviewers. We have attached a manuscript using track changes, and the line numbers we reference below refer to the track-changes version, as well as a “clean” revision. We document our changes in the letter below in red font, following reviewer comments, and are grateful to their comments which continued to improve our manuscript.

Thank you for the opportunity to revise our manuscript.

Sincerely,

Michelle D. Boone, Ph.D.	Melissa B. Youngquist, Ph.D.

Associate Professor	Research Biologist, Shedd Aquarium

PONE-D-21-04128R1
Larval development and survival of pond-breeding anurans in an agricultural landscape impacted more by phytoplankton than surrounding habitat
PLOS ONE

Journal Requirements:

Please review your reference list to ensure that it is complete and correct. If you have cited papers that have been retracted, please include the rationale for doing so in the manuscript text, or remove these references and replace them with relevant current references. Any changes to the reference list should be mentioned in the rebuttal letter that accompanies your revised manuscript. If you need to cite a retracted article, indicate the article’s retracted status in the References list and also include a citation and full reference for the retraction notice.

#1 We have not to our knowledge cited manuscripts that were retracted.

Additional Editor Comments:

Minor corrections should be finished.


[Note: HTML markup is below. Please do not edit.]

Reviewers' comments:

Reviewer's Responses to Questions

**Comments to the Author**

1. If the authors have adequately addressed your comments raised in a previous round of review and you feel that this manuscript is now acceptable for publication, you may indicate that here to bypass the “Comments to the Author” section, enter your conflict of interest statement in the “Confidential to Editor” section, and submit your "Accept" recommendation.

Reviewer #1: All comments have been addressed

Reviewer #2: All comments have been addressed

2. Is the manuscript technically sound, and do the data support the conclusions?

The manuscript must describe a technically sound piece of scientific research with data that supports the conclusions. Experiments must have been conducted rigorously, with appropriate controls, replication, and sample sizes. The conclusions must be drawn appropriately based on the data presented.

Reviewer #1: Yes

Reviewer #2: Yes

3. Has the statistical analysis been performed appropriately and rigorously?

Reviewer #1: Yes

Reviewer #2: Yes

4. Have the authors made all data underlying the findings in their manuscript fully available?

The [PLOS Data policy](http://www.plosone.org/static/policies.action#sharing) requires authors to make all data underlying the findings described in their manuscript fully available without restriction, with rare exception (please refer to the Data Availability Statement in the manuscript PDF file). The data should be provided as part of the manuscript or its supporting information, or deposited to a public repository. For example, in addition to summary statistics, the data points behind means, medians and variance measures should be available. If there are restrictions on publicly sharing data—e.g. participant privacy or use of data from a third party—those must be specified.

Reviewer #1: Yes

Reviewer #2: Yes

5. Is the manuscript presented in an intelligible fashion and written in standard English?

PLOS ONE does not copyedit accepted manuscripts, so the language in submitted articles must be clear, correct, and unambiguous. Any typographical or grammatical errors should be corrected at revision, so please note any specific errors here.

Reviewer #1: Yes

Reviewer #2: Yes

6. Review Comments to the Author

Please use the space provided to explain your answers to the questions above. You may also include additional comments for the author, including concerns about dual publication, research ethics, or publication ethics. (Please upload your review as an attachment if it exceeds 20,000 characters)

Reviewer #1: The authors have made substantial improvements to the manuscript clarifying several aspects of the methodology. As I have already said, the whole paper is very well written, the introduction builds logically to the hypotheses, and the discussion addresses the limitations of the experimental design in a way that does not weaken the conclusions that they were able to draw from the results.

I only have a couple of minor comments. See below (lines refer to the “clean” version of the text):

Line 202 – When you say, “generalized linear mixed models”: Just for clarity, since you could not fit the model with a binomial distribution and transformed the data to meet assumptions of normality, you could either say that used "linear mixed models" or "generalized mixed models with gaussian distribution".

#2 In the revision, MDB made a mistake in adding that survival was arcsine-square root transformed—this has been deleted from the revision. We clarified our methods regarding which distributions and model types were used for the various responses. (Lines 201-212)

Line 215 – Replace "We used pond was a random effect" with "We used pond as a random effect".”

#3 We made this change. (Line 223)

Lines 248 – 251: When you examine the effects of habitat type and pond origin on terrestrial growth and used the enclosure as the experimental unit, how exactly did get the values of your response variable? Did you average the final mass and change in mass within each enclosure?

#4 Yes, we averaged final mass and change in mass based on individuals within the enclosure to determine the enclosure mean. There were 18 enclosures, and 18 means for each response variable. We clarified the text. (Line 259)

Lines 252 – 23: When you examine the effects of initial mass on growth and survival, did you change the experimental unit from individuals to enclosures? Or did you keep individuals as the experimental unit as in the previous version? Please clarify that.

#5 We did use individuals in this analysis even though individuals were not reared separately to determine how size at metamorphosis impacted survival probability and future size. We clarified the text. (Lines 261-262)

Reviewer #2: The authors thoughtfully addressed the previous concerns with this manuscript and the discussion tells a much more cohesive story. Well done! Below are a few minor grammatical edits with one additional point to address.
Comments:
Nearly half the animals didn’t metamorphose in one of the forested ponds, any indication what was different with that pond? The response of survival to metamorphosis- did this exclude the number of tadpoles collected that hadn’t metamorphosed or are they counted as “dead”?

#6 Cricket frogs that did not reach metamorphosis were not included in “survival to metamorphosis”—this is the standard approach in amphibian ecology experiments. Using total survival (metamorphs plus tadpoles) did not change the conclusions made in this study. We are not sure why one pond had a larger number of tadpoles at the end of the study based on the responses we measured. (Lines 195-196)

Lines 208-211: Swap the order of results presented so it can read “one or two orders of magnitude, respectively”

#7 We made this change. (Lines 216-218)

Line 215: “was” should be “as”

#8 We made this change. (Line 223)

Line 323: remove comma after “use”

#9 We made this change. (Line 333)

Line 352: edit to “resource”

#10 We made this change. (Line 362)

While revising your submission, please upload your figure files to the Preflight Analysis and Conversion Engine (PACE) digital diagnostic tool, <https://pacev2.apexcovantage.com/>. PACE helps ensure that figures meet PLOS requirements. To use PACE, you must first register as a user. Registration is free. Then, login and navigate to the UPLOAD tab, where you will find detailed instructions on how to use the tool. If you encounter any issues or have any questions when using PACE, please email PLOS at [figures@plos.org](mailto:figures@plos.org). Please note that Supporting Information files do not need this step.

 #11 We did this.
